# The enhancement by arbuscular mycorrhizal fungi of the Cd remediation ability and bioenergy quality-related factors of five switchgrass cultivars in Cd-contaminated soil

**DOI:** 10.7717/peerj.4425

**Published:** 2018-03-06

**Authors:** Hong Sun, Yixiao Xie, Yulong Zheng, Yanli Lin, Fuyu Yang

**Affiliations:** 1 College of Animal Science and Technology, China Agricultural University, Beijing, China; 2 Beijing Sure Academy of Biosciences, Beijing, China

**Keywords:** *Rhizophagus irregularis*, Energy grass, Cellulose, Hemicellulose, Lignin, Biofuel, Bioethanol, Methane, Cd extrction

## Abstract

A greenhouse experiment was carried out to investigate the effects of arbuscular mycorrhizal fungi (AMF) on the growth, P and Cd concentrations and bioenergy quality-related factors of five cultivars of switchgrass, including three lowland cultivars (Alamo (Ala), Kanlow (Kan), Performer (Per)) and two highland cultivars (Blackwell (Bw), Summer (Sum)), with 0, 1 and 10 mg/kg Cd addition levels. The results showed that AMF inoculation notably increased the biomass and P concentrations of all the cultivars. The Cd concentrations in the roots were higher than those in the shoots of all cultivars irrespective of inoculation, but the AMF had different effects on Cd accumulation in highland and lowland cultivars. AMF inoculation decreased the shoot and root concentrations in Ala and Kan, increased the shoot and root concentrations of Cd in Bw and Sum, and increased shoot Cd concentrations and decreased root Cd concentrations in Per. The highest Cd concentrations were detected in the roots of Bw and in the shoots of Sum with AMF symbiosis. Bw contained the highest total extracted Cd which was primarily in the roots. Ala had the second highest extracted Cd in the shoots, reaching 32% with 1 mg/kg of added Cd, whereas Sum had the lowest extracted Cd. AMF symbiosis had varied effects on bioenergy quality-related factors: for example, AMF decreased the ash lignin content in Ala and the C/N in Sum, increased the nitrogen, gross calorie values, and maintained the hemicellulose and cellulose contents in all cultivars with all tested concentrations of Cd. A principal component analysis (PCA) showed that AMF inoculation could enhance, weaken or transform (positive-negative, PC1-PC2) the correlations of these factors with the principle components under Cd stress. Therefore, AMF symbiosis enhanced the growth of different cultivars of switchgrass, increased/decreased Cd accumulation, promoted Cd extraction, and regulated the bioenergy quality-related factors in Cd-polluted areas. Bw is a suitable cultivar for phytostabilization due to high root Cd stabilization, whereas Ala is an appropriate cultivar for phytoremediation of less polluted areas because of its high Cd extraction and excellent bioenergy quality.

## Introduction

With increasing levels of economic development, pollution with heavy metals is becoming a serious threat to the humans and environment, generally originating from industry, mining and agriculture (Jarup, 2002). Cadmium (Cd), the most toxic heavy metal contaminant, inhibits plant growth by destroying the photosynthetic structure, disturbs nutrition absorption, decreases root and leaf extension and even leads to death (Lin & Aarts, 2012; Zhang et al., 2014). Through the food chain, Cd can enter the animal and human body, damaging kidney and reproductive functions, and cause osteoporosis and hypertension (Peralta-Videa et al., 2009). Thus, remediation of Cd-polluted soil to avoid Cd damage to human health is an urgent problem. Compared with traditional methods, phytoremediation requires less money and labor and is more environmentally friendly; consequently it has become a promising alternative technology for using and restoring polluted soil (Marques, Rangel & Castro, 2009). However, most selected hyperaccumulator plants have low biomass and grow slowly, which restricts long-term utilization. Finding a plant species that can trade off the time cost and economic development in the phytoremediation process could be a favorable solution to phytoremediation.

Energy crops have been introduced to produce bioenergy (e.g., biofuels, biogas and bioethanol) as substitutes for traditional fossil fuels in recent decades (Lewandowski et al., 2003; Koçar & Civaş, 2013). With the decreasing area of cultivated land in the world, bioenergy grasses are expected to grow in abandoned or contaminated areas to simultaneously improve the ecological environment, produce biomass and inhibit the entrance of heavy metals (e.g., Cd) into the food chain (Zhang et al., 2014; Wang et al., 2015; Pandey, Bajpai & Singh, 2016). Until now, researchers have focused on the selection of energy grass based on their Cd tolerance and accumulation as well as their phytoremediation ability in simulated and real environments. These grasses include *Chrysopogon zizanioides*, *Arundo donax*, *Pennisetum americanum, Miscanthus sinensis*, *Elymus elongatus* subsp. and *Panicum virgatum* (Reed et al., 1999; Sabeen et al., 2013; Sipos et al., 2013; Ondo et al., 2014; Zhang et al., 2014; Firmin et al., 2015). Switchgrass *(Panicum virgatum*), a perennial warm season rhizomatous grass, originated in North America and can be used to produce biofuels, bioethanol and methane (Schmer et al., 2008; Koçar & Civaş, 2013; Wu et al., 2015; Kou et al., 2017). In addition, switchgrass has a broad climate tolerance, rapid growth rate, high biomass yield, low nutrition input requirements and strong tolerance of most abiotic and biotic stressors, which could be excellent advantages when using this grass as a phytoremediation tool (Lewandowski et al., 2003). Researchers have reported that switchgrass has a strong tolerance of water and salinity stress (Sánchez et al., 2016), but few studies have reported on its response to metal stress. Although all relevant studies found that switchgrass was Cd-tolerant, the dramatic inhibition of switchgrass growth by Cd toxicity could not be ignored (Reed et al., 1999; Chen et al., 2011). Due to differences in morphology, switchgrass is categorized into two broad types, upland and lowland (Porter, 1966). To select a proper cultivar and find a rapid solution to increase switchgrass biomass to compensate for the low Cd accumulation in Cd-contaminated soil were urgent problems.

Most previous studies have focused on the ability of plants to extract or stabilize contaminants, but attention should be paid to utilizing harvested biomass materials because mishandled materials can easily cause secondary pollution and a waste of resources. Planting grasses as energy crops in polluted areas might not only prevent heavy metal from entering the food chain but also replace traditional fossil fuels with the harvested biomass materials to produce heat, methane and bioethanol. The gross calorie value (GCV) and the ash and alkali metal contents indicate the properties of fuels. The C/N shows the potential ability of biomass to produce methane via microorganisms, and the amounts of hemicellulose, cellulose and lignin indicate the potential for the production of bioethanol from biomass materials (Jarchow et al., 2012; Wu et al., 2015; Kou et al., 2017). However, there have been few studies about the biomass quality of grass energy crops harvested from soils polluted with heavy metals. Since grass with strong heavy metal phytoremediation ability must be accompanied by lower biomass and bioenergy quality, such as higher ash content, it is critical to discover how to maintain or improve those qualities.

Arbuscular mycorrhizal fungi (AMF) are a key functional group of soil microbes that form symbiotic relationships with 80% of vascular plant species on land, and play a positive role in improving plant biomass and tolerance to abiotic stress by improves plant nutrition absorption, especially phosphorus (P). Recently, there have been many studies concerning the potential use of plants inoculated with AMF in contaminated soils. AMF symbiosis could improve immobilize contaminants in plant roots, extraradical mycelium and the mycorrhizal structure, reduce their directly hurt to above organisms of plants (Yao et al., 2014; Nayuki et al., 2014; Li et al., 2016). In addition, some studies have found with AMF symbiosis accelerating Cd translocation from roots to shoots and simultaneously enhancing plant growth, by the dilutive effect indirectly diminish the contaminant damages (Liu et al., 2015). These variations in results might result from the functional diversity in the symbioses between different AMF strains and various plant species (Zhang, Chen & Ohtomo, 2015; Gu et al., 2017). There have been many studies concerning the potential use of energy grass inoculated with AMF in contaminated soils. Recently, researchers have found AMF symbiosis could promote plant growth, improve heavy metal extraction or stabilization in several bioenergy grasses, as *Chrysopogon zizanioides*, *Miscanthus* × *giganteus*, *Festuca arundinacea*, *Lolium perenne* (Bahraminia et al., 2016; Firmin et al., 2015; Gu et al., 2017). The studies about switchgrass inoculated AMF were few. To date, researchers reported that inoculation with AMF improves switchgrass growth in acidic soil and helps switchgrass to use more nitrogen with increasing temperature (Clark, Zeto & Zobel, 1999; Schroeder-Moreno et al., 2011). Only Arora, Sharma & Monti (2016) reported that AMF symbiosis decreased Cd translocation index in one cultivar of switchgrass over time, but it is unknown whether AMF symbiosis had the same effects on other switchgrass cultivars under conditions of Cd addition. In addition, there have been few studies about the effect of AMF on energy grass bioenergy quality, regardless of soil contamination, other than a study by Liu et al. that (2014) found that poplar, a bioenergy woody plant with a fast growth rate and short rotation cycle, when inoculated with AMF had higher GCV, organic carbon and lignin than non-inoculated plants. Hence, there is practical significance to exploring the bioenergy quality of bioenergy grass with AMF symbiosis, especially under heavy metal stress.

Therefore, the objective of this experiment was to investigate the effects Cd addition on the growth, Cd accumulation, and bioenergy quality related to the production of methane (carbon, nitrogen, C/N), bioethanol (hemicellulose, cellulose, lignin content) and combustion (ash, GCV, alkalis content) of five cultivars with and without AMF symbiosis. The present study focused on selecting the cultivar with the highest Cd extraction and optimum bioenergy quality to provide a basis for supporting the application of switchgrass inoculated with AMF as a strategy to phytoremediate Cd-polluted soils.

## Materials and Methods

### Growth media preparation

Soil was collected from the Shangzhuang Experiment Station of China Agricultural University. The soil chemical properties were as follows: pH 8.27 (1:1 in water), 50.56 mg/kg available N, 15 mg/kg available P, 118.96 mg/kg available K, 1.13% soil organic matter, 0.27 mg/kg total cadmium. The soil was air dried and passed through a 2-mm mesh sieve. Sand was bought from the Dasanlan Garden material station (diameter <0.5 mm), washed several times, and then rinsed three times in deionized water. The soil and sand were mixed uniformly (w/w = 2:1) and sterilized by gamma rays (25kGy, 10 MeVelecton beam). Then, the mixtures were divided into three parts, and Cd (in the form of CdCl_2_.5H_2_O) was added at three levels, 0 mg/kg, 1 mg/kg, and 10 mg/kg standing for 50 days. The 1 mg/kg Cd was designed as the low Cd level, which is the highest critical value for agricultural and forestry production and the normal growth of plants as set by the Chinese Environmental Quality Standard for Soils (GB 15618-1995). The 10 mg/kg was set as the high Cd level, which was reported to affect switchgrass growth in a previous study (Chen et al., 2011). The soil medium was added with basal nutrition of 30 mg/kg P, 120 mg/kg N, and 120 mg/kg K.

### Host plants

The seeds of five cultivars of switchgrass (Ala, Kan, Per, Bw and Sum) were provided by the Breeding Laboratory of the Animal Science and Technology College of China Agricultural University. The seeds were sterilized in 10% H_2_O_2_ for 20 min, washed with tap water, and then rinsed three times with deionized water. Then, the seeds germinated until the radicles reached 3 cm before being planted in sterilized soil and grown for two months. Then, the similar growth condition seedlings for each cultivar were harvested for transplantation after being cut into 10-cm pieces for the aboveground portions and 3 cm pieces for the belowground portions.

### AMF preparation

The *Rhizophagus irregularis* (RI, BGC AH01) was provided by Baodong Cheng from research center for Eco-Environmental Sciences of Chinese academy of sciences. The harvested inoculum were consisted with a mixture of spores, hyphae and plant root fragment. One gram incolum included 200 spores.

### Pot experiment

The experiment was designed with three Cd levels (0, 1, 10 mg/kg Cd), two AMF treatments (inoculation or no inoculation with RI and five cultivars of switchgrass. Each treatment was replicated three times. A total of 90 pots were maintained.

Soil (1.2 kg) was placed into each plastic pot (*φ* 19 cm × height 17 cm). When the pot was two-thirds full, 25 g of the inoculum was added uniformly, then covered with the remaining soil. Then, two harvested seedlings were transplanted into each pot. The inoculum was sterilized at 121 °C for 4 h to create the uninoculated treatment. To keep the same soil bacteria except AMF, the uninoculated treatment was supplied with 5 ml of filtrate that was passed through a 40-mesh sieve. The pots were watered with deionized water to maintain a moisture content of 15% on a dry soil basis (55% water holding capacity). The experiment was conducted in a greenhouse with 20 h/30 °C days and 8 h/24 °C nights under natural light.

### Harvest and sample analysis

Three month later, all the plants were harvested and separated by shoot and root. The roots were washed by tap water and deionized water carefully. The fresh roots were divided into two parts, the fresh part was used to determine colonization, and the rest parts were used to determine the dry weight with shoot part under 65 °C for 48 h. A total of 1 cm fine root fragments were collected from every cultivar roots for each replicate to determine the rate of colonization by the grid line intersect method under stained with aniline blue. The dried material was milled and digested by HNO_3_ and H_2_O_2_ (3:1 v/v) in a microwave-accelerated reaction system (MarsX; CEM, USA) with a three-step digestion process. The concentrations of Cd, P, Ca, K, Na and Mg were determined by inductively coupled plasma-mass spectrometry (ICP-MS; model 7500a; Agilent Technologies, Santa Clara, CA, USA). Half a gram of 100-mesh fine powder of the dried material was used to determine the ash content, which was ashed in a muffle furnace for 5 h. One milligram of 100-mesh fine powder of the dried material was used in the determination of the total carbon and total nitrogen using an elemental analyzer (EA1108; Carlo Erba Strumentazione, Milan, Italy). One gram of 100-mesh fine powder of the dried material was used for the GCV determination using an automatic oxygen bomb calorimeter (6400, Parr Instrument Company, USA).

Hemicellulose was calculated as neutral detergent fiber (NDF) minus acid detergent fiber (ADF), and cellulose was calculated as ADF minus acid detergent lignin (ADL). Lignin (mainly referring to acid-insoluble lignin) was calculated as the ADL value minus ash. The NDF, ADF and ADL values were determined according to the Ankom Filter Bag method.

### Statistical analysis

The Cd extraction amount was used to evaluate Cd phytoextraction from soil and was calculated using Eq. (1). The responses of growth (%MGR), P concentration (%MPR), and Cd concentration (%MCdR) to mycorrhizal inoculation were used to show the effects of AMF on biomass, P and Cd concentrations compared with the non-inoculated treatment and were calculated using Eq. (2):

(1) \begin{eqnarray*}& & \text{Cd extracted amount}=\text{Cd concentration}\times \text{biomass of per pot}\end{eqnarray*}

(2) \begin{eqnarray*}& & \text{%}\mathrm{MXR}= \frac{{X}_{\mathrm{AM}}-{X}_{\mathrm{NM}}}{{X}_{\mathrm{NM}}} \times 100\end{eqnarray*}

where *X*_AM_ is biomass or the P or Cd concentrations in AM plants, and *X*_NM_ represents the average biomass, the P or Cd concentrations in non-AMF plants at every Cd level, respectively.

The biomass, P and Cd concentrations, ash content, total C, total N, C/N, GCV, cellulose, hemicellulose and lignin content and K, Na, Ca, Mg concentrations response data were subjected to a two-way analysis of variance (ANOVA) to test the significance of the Cd addition, inoculation treatment and the cultivar. The data were analyzed with SPSS 17.0. The means of biomass, P and Cd concentrations and bioenergy-factors under the same Cd levels were examined for significant differences by Duncan’s multiple range test.

A PCA was performed to determine the correlations among bioenergy quality-related factors. The correlation variations were compared between inoculated and non-inoculated treatments in the five cultivars to identify the effects of AMF inoculation after the addition of three concentrations of Cd. Canoco for Windows 4.5 was used to analyze the data. In the software, we selected the three Cd concentrations as the sample variables, the Cd level in the shoots as the environment variable, and the C, N, C/N, hemicellulose, cellulose, lignin, ash, GCV and alkali elements as the species variables. We obtained average values of each Cd concentration for every cultivar.

## Result

### Biomass

**Figure 1:**
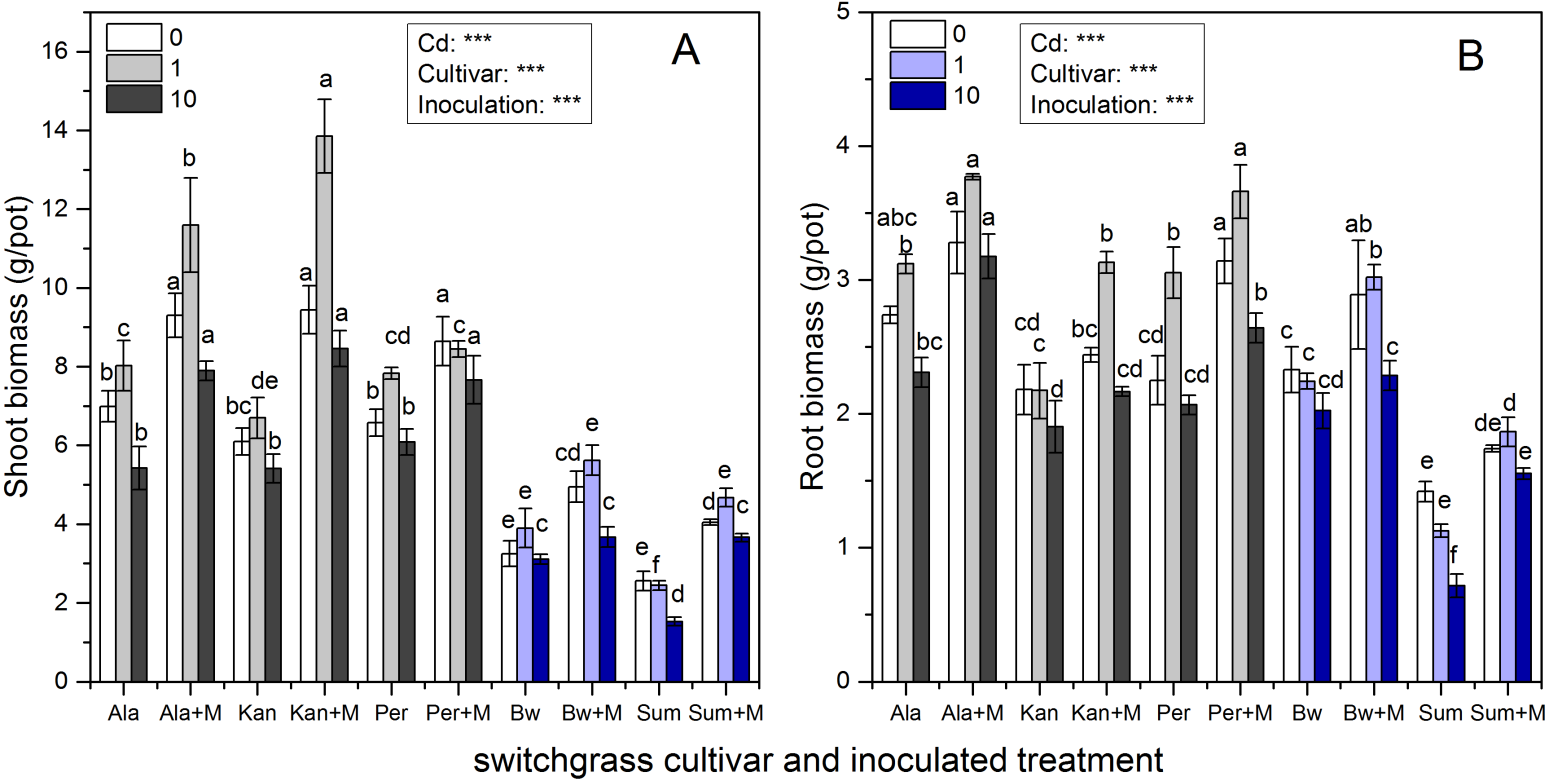
The biomass in the shoots (A) and roots (B) of five switchgrass cultivars with the NM (non-inoculated with AMF) and M (inoculated with AMF, +M) treatments under 0, 1 and 10 mg/kg Cd addition. The letters above the bar represent the significantly difference at 0.05 levels under the same Cd addition level of the five cultivars, regardless of inoculation.

Figure 1 shows that the shoot and root biomass of the five cultivars were significantly affected by inoculation, Cd addition and their interaction (*P* < 0.001), except for their interaction affecting root biomass (*P* > 0.05). The shoot biomass of the three lowland cultivars (Ala, Kan and Per) were much higher than those of the two highland cultivars (Bw and Sum), irrespective of inoculation or Cd addition level (*P* < 0.05), whereas the root biomass of Bw was similar to those of the three lowland cultivars under the three Cd levels (*P* > 0.05). As soil Cd increased, shoot biomass tended to increase with low Cd concentration (1 mg/kg) and then decrease when the soil Cd reached 10 mg/kg for Ala, Kan, and Bw irrespective of inoculation and Per without AMF. The same trend was found for the root biomass of Ala and Per irrespective of inoculation and Kan, Bw with AMF. Both the shoot and root biomass of S without AMF inoculation decreased with increasing soil Cd.

AMF significantly promoted shoot and root biomass for all cultivars, while the range in increases observed was quite distinct. The biomass of Ala with RI was higher than that of non-inoculated at all Cd levels. Ala biomass increased by 33%, 45%, and 46% in the shoots and 20%, 21%, and 38% in the roots under 0, 1 and 10 mg/kg Cd concentrations, respectively. Compared with the non-inoculated treatment, the inoculated Kan shoot biomass was promoted by 55%, 107% and 56% for shoots and by 12%, 44% and 14% for roots with 0, 1 and 10 mg/kg Cd concentrations, respectively. For Per, the shoot biomass increased with AMF symbiosis by 31%, 8%, and 26%, and the root biomass increased by 40%, 20%, and 28% under 0, 1 and 10 mg/kg Cd concentrations, respectively. The shoot biomass of inoculated Bw and Sum was increased by 52%, 44%, and 18% and by 59%, 91%, and 140% compared with the non-inoculated treatment with 0, 1 and 10 mg/kg Cd concentrations, respectively. The corresponding root biomass increased by 24%, 35%, and 13% in Bw and by 23%, 66%, and 117% in Sum with 0, 1 and 10 mg/kg Cd concentrations, respectively.

### P concentration

**Figure 2:**
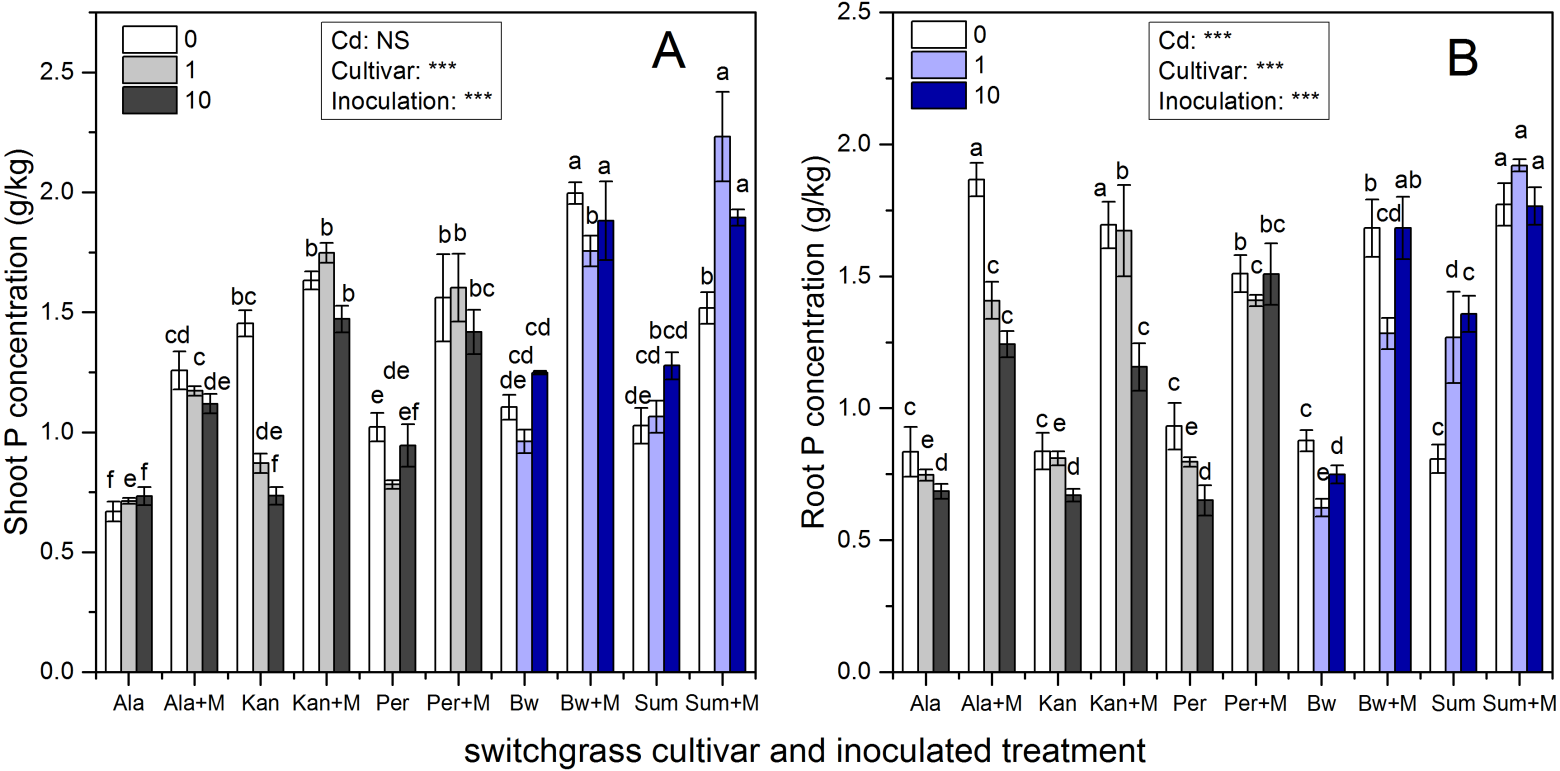
The P concentrations in the shoots (A) and roots (B) of five switchgrass cultivars with NM and M treatment under 0, 1 and 10 mg/kg Cd addition. The letters above the bar represent the significantly difference at 0.05 levels under the same Cd addition level of the five cultivars, regardless of inoculation.

Figure 2 presents the shoot and root P concentrations of the five cultivars, which were significantly affected by inoculation, Cd addition and their interaction (*P* < 0.05, *P* < 0.01, *P* < 0.001, respectively), except for Cd addition on shoot P concentrations (*P* > 0.05). AMF symbiosis greatly enhanced the P concentrations in the shoots and roots of the five cultivars irrespective of Cd addition levels (*P* < 0.05).

The ability of RI to promote P absorption in different switchgrass cultivars varied. The P concentrations of Ala with RI were higher than those for non-inoculated Ala at all Cd levels, which increased by 88%, 64%, and 53% in the shoots and by 124%, 89%, and 81% in the roots in 0, 1 and 10 mg/kg Cd soils, respectively. In addition, for Kan, AMF promoted P concentrations by 12%, 101% and 100% in the shoots and by 100%, 107% and 73% in the roots in 0, 1 and 10 mg/kg Cd soils, respectively. For Per, the shoot P concentration increased with AMF symbiosis by 53%, 104%, and 49%, and the root P concentration increased by 62%, 77%, and 132% with the three increasing Cd additional levels. The shoot P concentration of inoculated Bw and Sum increased by 81%, 83%, and 51% in Bw and by 48%, 110%, 49% in Sum compared with non-inoculated plants in 0, 1 and 10 mg/kg Cd soils, respectively. Their corresponding root P concentrations increased by 92%, 106%, and 125% in Bw and by 120%, 51%, and 30% in Sum with the three increasing Cd additional levels.

### Cd concentration

**Figure 3:**
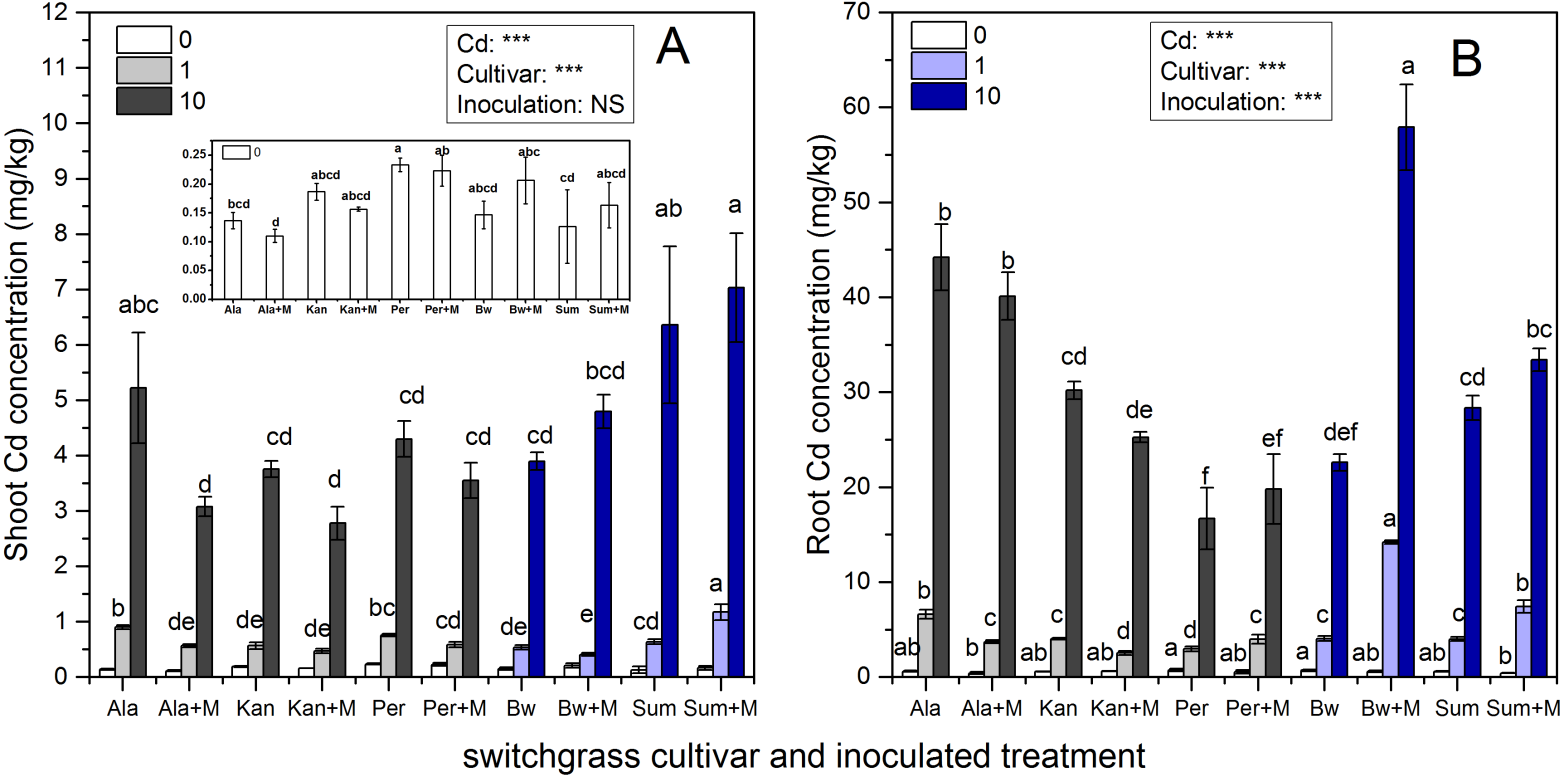
The Cd concentrations in the shoots (A) and roots (B) of five switchgrass cultivars with NM and M treatment under 0, 1 and 10 mg/kg Cd addition. The letters above the bar represent the significantly difference at 0.05 levels under the same Cd addition level of the five cultivars, regardless of inoculation.

Cd concentrations in the shoots and roots of the five cultivars with the three Cd addition levels with or without AMF symbiosis are shown in Fig. 3. AMF inoculation, Cd addition and their interaction significantly affected the Cd concentrations of the five cultivars in the shoots and roots (*P* < 0.05; *P* < 0.01, *P* < 0.001), while their interaction did not affect the Cd concentration in Ala or Per (*P* > 0.05). Significant decreases in Cd concentrations were observed in the shoots of Ala and in the roots of Ala and Kan under 1 mg/kg inoculated with RI; however, the Cd enhancement effects were with RI symbiosis observed in shoots of Sum under 1 mg/kg Cd addition, and the roots of Per, Bw and Sum under 1 mg/kg and Sum under 10 mg/kg Cd treatments (*P* < 0.001, *P* < 0.05).

As the concentration of Cd in the soil increased, the Cd concentration in the shoots and roots significantly increased in all cultivars irrespective of inoculation with AMF, although the Cd concentrations in different cultivars varied with AMF inoculation. The Cd concentration in shoots of Sum and roots of Bw with AMF inoculation were higher than other cultivar under 1 and 10 mg/kg Cd addition, respectively.

The increases and decreases in Cd concentrations due to AMF symbiosis varied in different cultivars. The Cd concentrations in the shoots of Ala and Kan inoculated with AMF decreased by 37% and 17%, respectively, in 1 mg/kg Cd soils, and by 41%% and 26%, respectively, in 10 mg/kg Cd soils. The Cd concentration in the roots of Ala and Kan decreased by 44% and 38%, respectively, in 1 mg/kg Cd soils and by 13% and 16%, respectively, in 10 mg/kg Cd soils. The Cd concentrations in the shoots of Per were decreased by 23% and 18% with 1 and 10 mg/kg Cd soil, respectively, and the Cd concentrations in inoculated Bw decreased by 23% compared with non-inoculated Bw with the addition of 1 mg/kg of Cd. However, except for the roots of Bw at the 1 mg/kg Cd level, the presence of AMF significantly promoted Cd accumulation in the shoots of Sum, which increased by 84%, respectively, with the addition of 1 mg/kg of Cd and by 10% and 22%, respectively, with the addition of 10 mg/kg of Cd. Cd accumulation was also promoted in the roots of Sum and Bw, increasing by 85% and 252%, respectively, with the addition of 1 mg/kg of Cd and by 18% and 156%, respectively, with the addition of 10 mg/kg of Cd. The root Cd concentrations of Per were increased by 35% and 19% in both inoculated and non-inoculated treatments with the addition of either 1 or 10 mg/kg of Cd.

### Cd extraction

**Figure 4:**
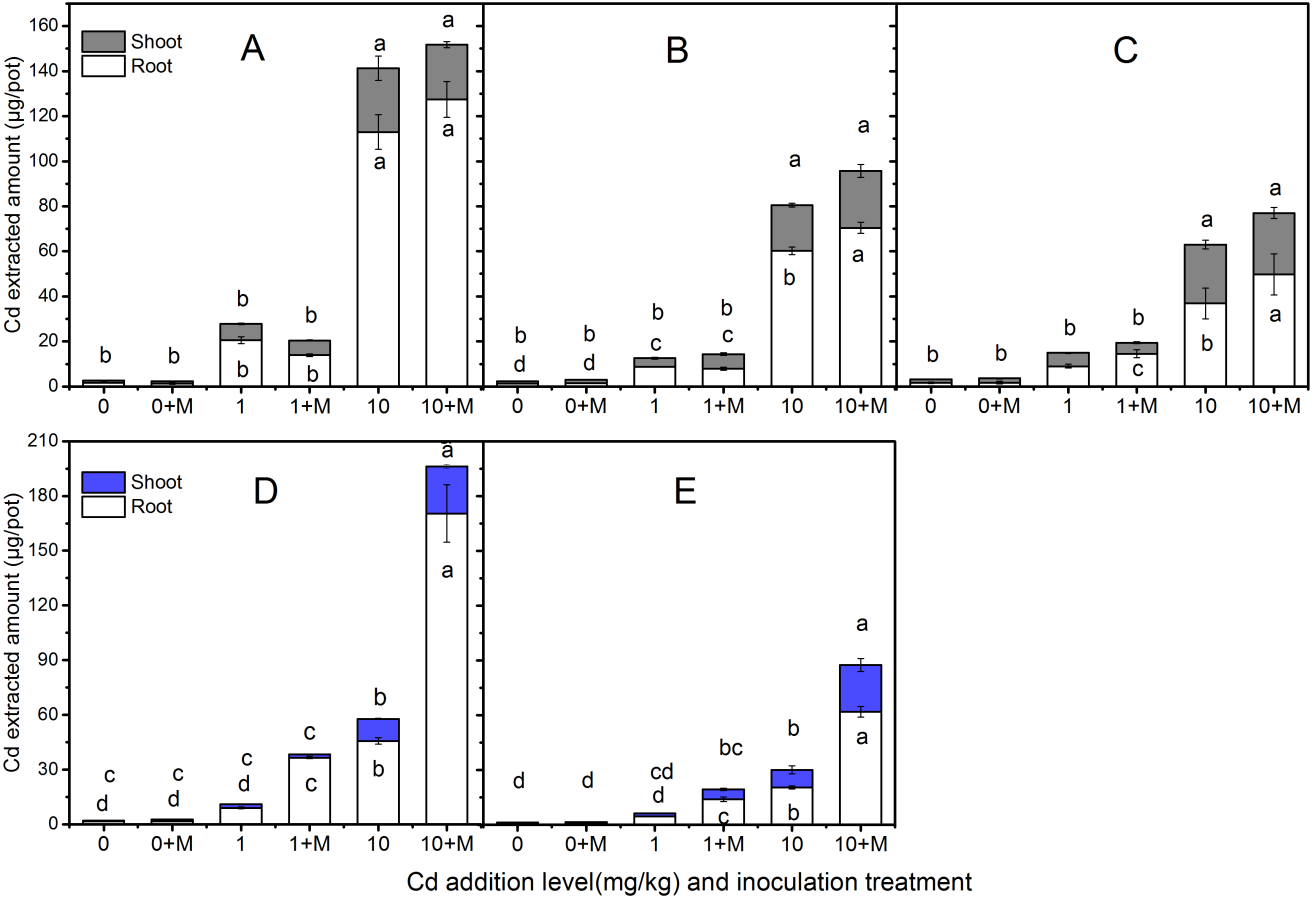
The Cd extracted amount in the shoots and roots of five cultivars with NM and M treatment under 0, 1 and 10 mg/kg Cd addition. The letters above the bar represent the significantly difference at 0.05 levels under three Cd levels, regardless of inoculation. The bars with one letter means shoot and root having the same letter. (A–E) refer to Ala, Kan, Per, Bw, Sum, respectively.

The extracted Cd amount in the shoots and roots of the five cultivars were shown in Fig. 4 which increased with increasing soil Cd. Nevertheless, the Cd extracted amount were diverse. The trends in shoot extracted Cd amount were: Ala, Kan with AMF; Ala > Per, Sum with AMF; Per  > Kan > Bw (AMF absence or presence), Sum under 1 mg/kg Cd additional level; Ala, Per (AMF absence or presence); Kan, Bw, Sum with AMF > Kan > Bw and Sum in 10 mg/kg Cd soil. In addition, the root Cd extrction trend in 1 mg/kg Cd soil was: Bw with AMF > Ala > Ala, Per and Sum with AMF > Kan with AMF symbiosis; Kan, Per, Bw > Sum; and in 10 mg/kg Cd soil, the trend was: Bw with AMF > Ala (AMF absence or presence) > Kan (AMF absence or presence); Per, Sum with AMF > Per, Bw > Sum.

No significant differences were found in shoot Cd extraction between inoculated and non-inoculated treatments in the three lowland cultivars at the three Cd addition levels (*P* > 0.05), except the Cd extraction in Ala and Per under 10 mg/kg Cd level (*P* < 0.05). However, compared with the non-inoculated treatment, AMF helped Per, Bw, and Sum extract more Cd in their roots, increasing by 61%, 303%, and 207% , respectively, in 1 mg/kg Cd soils, and Ala, Kan, Per, Bw and Sum by 13%, 17%, 35%, 273% and 204%, respectively, in 10 mg/kg Cd soils. Moreover, the Cd extraction with AMF symbiosis was higher than that in the non-inoculated treatment under 10 mg/kg Cd addition in the shoots of Bw and Sum, which increased by 113% and 164%, respectively. The inoculated corresponding Cd extraction under 10 mg/kg Cd addition in the shoots of Ala, Per and Bw decreased by 10%, 17%, and 33% compared with non-inoculated treatment, respectively.

### Mycorrhizal response

The mycorrhizal responses to biomass (%MBR), P concentration (%MPR) and Cd concentration (%MCdR) of the five cultivars at the three Cd addition levels are shown in Table 1. AMF symbiosis had positive responses to switchgrass biomass and P concentration and negative responses to Cd concentration in the lowland cultivars. The %MBR of the five cultivars showed a different trend. With increasing Cd level, the %MBR in the shoots and roots of Ala and Sum increased, those in Kan and Bw increased initially but then decreased, and that in Per decreased. With increasing Cd, the %MPR in the shoots of Ala and Bw decreased, while it increased in the shoots of Kan and Per and in the roots of Per and Bw; in addition, the %MPR in the roots of Kan and in the shoots and roots of Sum increased with the addition of 1 mg/kg Cd and then decreased with the addition of 10 mg/kg. The %MCdR showed a decreasing trend as soil Cd rose, except in the shoots of Ala and Kan.

**Table 1:** The mycorrhizal response to biomass (MBR%), P concentration (MPR%) and Cd concentration (MCdR%) of five cultivars under three Cd addition levels.

Cultivar	Cd level	MBR%		MBR%		MBR%	
		Shoot	Root	Shoot	Root	Shoot	Root
Ala	0	33 ± 8	20 ± 8	88 ± 11	124 ± 8	−18 ± 9	−35 ± 20
	1	45 ± 15	21 ± 7	64 ± 3	89 ± 9	−37 ± 3	−44 ± 2
	10	46 ± 5	38 ± 7	52 ± 5	81 ± 7	−41 ± 3	−9 ± 6
	0	55 ± 10	12 ± 2	12 ± 3	100 ± 10	−16 ± 2	7 ± 4
Kan	1	107 ± 14	44 ± 4	101 ± 5	106 ± 21	−17 ± 7	−38 ± 5
	10	56 ± 8	14 ± 2	100 ± 8	73 ± 14	−26 ± 8	−16 ± 2
	0	31 ± 9	40 ± 7	53 ± 18	62 ± 8	−3 ± 12	−26 ± 25
Per	1	8 ± 3	20 ± 7	105 ± 18	77 ± 3	−23 ± 7	35 ± 16
	10	26 ± 10	28 ± 5	50 ± 10	132 ± 18	−17 ± 7	19 ± 22
	0	52 ± 12	24 ± 17	81 ± 4	92 ± 12	41 ± 28	−17 ± 13
Bw	1	44 ± 10	35 ± 4	82 ± 7	106 ± 9	−23 ± 6	252 ± 5
	10	18 ± 8	13 ± 6	51 ± 13	125 ± 16	23 ± 8	156 ± 20
	0	59 ± 3	23 ± 2	48 ± 6	119 ± 10	27 ± 32	−27 ± 4
Sum	1	91 ± 9	66 ± 10	110 ± 18	51 ± 2	84 ± 22	85 ± 17
	10	140 ± 7	117 ± 6	48 ± 3	30 ± 5	10 ± 15	18 ± 4

### C, N, C/N, hemicellulose, cellulose, lignin

The contents of C, N, C/N, hemicellulose, cellulose, and lignin of the five cultivars of switchgrass under different Cd addition levels are provided in Table 2. The contents of C, N, C/N, hemicellulose were significantly influenced by Cd addition, and the contents of C, N, C/N, were significantly affected by inoculation with AMF (*P* < 0.05, *P* < 0.01, *P* < 0.001, Table S1); the interaction between Cd addition and AMF inoculation significantly influenced the N content (*P* < 0.05, Table S1). In addition, the cultivar significantly affected all the indexes of bioenergy quality (*P* < 0.05, *P* < 0.01, *P* < 0.001, Table S1).

**Table 2:** The Content of C, N, C/N, hemicellulose, cellulose and lignin in five cultivars with the NM and M treatment under three Cd concentrations. The letters in the same column under the same Cd addition level indicate significant differences at *P* < 0.05 among five cultivars, regardless of inoculation.

Cd level (mg/kg)	Cultivar		C (%)	N (%)	C/N	Hemicellulose (%)	Cellulose (%)	Lignin (%)
**0**	Ala	NM	43.9 ± 0.0bc	1.44 ± 0.02bc	30.5 ± 0.4ab	29.9 ± 0.3ab	31.4 ± 0.1ab	5.3 ± 0.1ab
		M	44.1 ± 0.2ab	1.33 ± 0.06c	33.3 ± 1.5a	30.3 ± 0.6ab	29.2 ± 0.4c	4.4 ± 0.3abc
	Kan	NM	44.1 ± 0.1ab	1.56 ± 0.08abc	28.5 ± 1.6bc	31.8 ± 0.8a	31.4 ± 0.7ab	4.2 ± 0.2bc
		M	43.9 ± 0.0bc	1.65 ± 0.05ab	26.7 ± 0.9bc	31.8 ± 1.1a	32.0 ± 0.3a	5.2 ± 0.7abc
	Per	NM	44.4 ± 0.1a	1.67 ± 0.09ab	26.7 ± 1.4bc	30.8 ± 0.6ab	31.5 ± 0.0ab	4.3 ± 0.3abc
		M	44.3 ± 0.1ab	1.66 ± 0.01ab	26.6 ± 0.2bc	30.9 ± 1.0ab	30.4 ± 1.2abc	4.6 ± 0.8abc
	Bw	NM	43.4 ± 0.1d	1.52 ± 0.02abc	28.6 ± 0.4bc	29.5 ± 0.2b	31.7 ± 0.3ab	5.4 ± 0.5a
		M	440.± 0.2b	1.74 ± 0.07a	25.4 ± 1.1c	30.6 ± 0.6ab	30.0 ± 0.9abc	4.1 ± 0.4c
	Sum	NM	43.6 ± 0.3cd	1.57 ± 0.10abc	27.9 ± 1.5bc	31.0 ± 0.4ab	29.7 ± 1.0bc	4.5 ± 0.1abc
		M	44.2 ± 0.2ab	1.65 ± 0.18ab	27.5 ± 3.0bc	31.0 ± 0.7ab	28.7 ± 0.8c	4.7 ± 0.1abc
**1**	Ala	NM	44.3 ± 0.1bc	1.27 ± 0.04b	34.9 ± 1.0a	31.2 ± 0.1abc	31.1 ± 0.9a	4.9 ± 0.2a
		M	44.2 ± 0.1bc	1.34 ± 0.01b	32.9 ± 0.4ab	30.3 ± 0.7bc	30.5 ± 0.2a	4.3 ± 0.2de
	Kan	NM	44.5 ± 0.1b	1.45 ± 0.04b	30.7 ± 0.9bc	31.5 ± 0.6abc	32.3 ± 0.5a	4.4 ± 0.2cde
		M	44.4 ± 0.2bc	1.56 ± 0.02ab	28.4 ± 0.4cd	32.3 ± 0.3a	30.7 ± 0.3a	4.8 ± 0.1ab
	Per	NM	44.4 ± 0.2b	1.51 ± 0.09b	29.6 ± 1.9bc	31.7 ± 0.3ab	30.7 ± 1.5a	4.1 ± 0.1e
		M	44.8 ± 0.1a	1.70 ± 0.12a	26.7 ± 2.0d	31.7 ± 0.6ab	30.2 ± 1.4a	3.6 ± 0.2f
	Bw	NM	44.1 ± 0.0c	1.41 ± 0.09b	31.4 ± 2.2abc	30.8 ± 0.4abc	30.7 ± 0.7a	4.4 ± 0.1bcde
		M	44.1 ± 0.1c	1.60 ± 0.02ab	27.6 ± 0.3cd	30.1 ± 0.3c	30.9 ± 0.8a	4.7 ± 0.1abcd
	Sum	NM	44.4 ± 0.0bc	1.57 ± 0.08b	28.4 ± 1.5cd	31.7 ± 0.4ab	31.1 ± 0.5a	4.5 ± 0.2abcde
		M	43.7 ± 0.1d	1.75 ± 0.04a	25.0 ± 0.5d	31.5 ± 0.2abc	30.3 ± 0.7a	4.8 ± 0.1abc
**10**	Ala	NM	43.6 ± 0.1c	1.27 ± 0.02e	34.3 ± 0.5a	30.6 ± 0.6a	29.9 ± 0.6d	5.0 ± 0.3a
		M	43.8 ± 0.2c	1.33 ± 0.05de	33.1 ± 1.4a	31.2 ± 0.4a	31.3 ± 0.4abcd	3.6 ± 0.3b
	Kan	NM	44.4 ± 0.1a	1.42 ± 0.03cd	31.4 ± 0.7abc	31.2 ± 0.4a	32.9 ± 0.4a	5.0 ± 0.2a
		M	44.6 ± 0.1a	1.36 ± 0.05de	32.8 ± 1.1ab	31.9 ± 0.7a	32.3 ± 0.3ab	4.3 ± 0.3ab
	Per	NM	44.3 ± 0.3ab	1.54 ± 0.06bc	28.9 ± 1.3cd	31.1 ± 0.1a	31.9 ± 0.6abc	4.0 ± 0.3b
		M	44.3 ± 0.1ab	1.65 ± 0.05ab	26.9 ± 0.9de	32.1 ± 0.4a	32.3 ± 0.7ab	4.0 ± 0.4b
	Bw	NM	43.7 ± 0.1c	1.53 ± 0.03bc	28.6 ± 0.6cde	30.9 ± 1.6a	29.6 ± 0.6d	4.4 ± 0.4ab
		M	43.8 ± 0.3bc	1.60 ± 0.05ab	27.4 ± 0.9de	31.0 ± 0.7a	30.9 ± 1.0bcd	4.3 ± 0.2ab
	Sum	NM	42.8 ± 0.0d	1.44 ± 0.07cd	29.8 ± 1.5bc	30.8 ± 0.1a	30.6 ± 0.6bcd	4.9 ± 0.2a
		M	43.8 ± 0.2b	1.71 ± 0.04a	25.6 ± 0.8e	32.1 ± 0.7a	30.2 ± 0.6cd	4.3 ± 0.3ab

The C contents in Bw and Sum with AM symbiosis were higher than those in the non-inoculated treatment and increased by 1.4% and 1.4%, respectively, with 0 mg/kg Cd. The same C content promotion effect by AMF was observed in Per with 1 and 10 mg/g Cd, which were increased by 0.9% and 0.1% compared with non-inoculation, respectively. The N contents in Ala were the lowest, irrespective of Cd concentration or inoculation. AM symbiosis improved the N contents in Ala, Kan, Per, Bw, Sum by 5.5%, 7.8%, 12.6%, 13.0%, 11.2% at the 1 mg/kg Cd addition level and by 4.7%, −3.8%, 7.4%, 4.8%, 18.5% at the 10 mg/kg Cd addition level, respectively, although most of these changes were not significantly different between inoculated and non-inoculated treatments. The C/N values in Ala were the highest at every Cd addition level, irrespective of AMF presence or absence and Cd level. Except C/N in Ala under 0 mg/kg and Kan under 10 mg/kg, the C/N in all inoculated cultivars were decreased compared with non-inoculated under all Cd addition levels.

The hemicellulose contents in Kan, Per and Sum were higher than those of Ala and Bw at the 0 mg/kg Cd additional level, whereas there were no significant differences between treatments with and without AMF. The cellulose contents of Ala, Per, Bw and Sum with AMF symbiosis was lower than that of the corresponding non-inoculated treatments in 0 mg/kg Cd soils, decreasing by 7.3%, 3.7%, 5.5% and 3.6%, respectively. In addition, the cellulose contents of Ala and Bw with RI increased by 4.6% and 4.3%, respectively, compared with those of the non-inoculated treatments in 10 mg/kg Cd addition level, although they were not significantly different. The lignin contents of Ala and Bw with AMF decreased by 17.2% 24.5%, respectively, compared with those of the non-inoculated treatments in 0 mg/kg Cd soil. Although AMF symbiosis decreased the lignin contents of Ala, Per by 11.6%, 13.7% with 1 mg/kg Cd addition. The lignin content of Ala and Kan greatly decreased by 27.8% and 15.2%, respectively, in 10 mg/kg Cd soil with AMF symbiosis, while the other cultivars were not significantly different between inoculated and non-inoculated treatments.

### Ash, GCV, Alkali metal concentration

The contents of ash, GCV, K, Na, Ca and Mg were shown in Table 3. The concentrations of ash, GCV, Na, Ca and Mg in the five cultivars were significantly affected by inoculation, Cd addition and their interactions, except the effects of inoculation on ash content, Cd addition on Mg nor they interaction on ash and Mg (*P* < 0.001, *P* > 0.05, Table S1). There was no significant difference between AMF presence and absence for the K concentrations of the five cultivars at the three Cd levels (*P* > 0.05, Table S1).

**Table 3:** The content of Ash, GCV, K, Na, Ca and Mg of five cultivar switchgrass with the NM and M treatment under three Cd concentrations. The letters in the same column under the same Cd addition level indicate significant differences at *P* < 0.05 among five cultivars, regardless of inoculation.

Cd level	Cultivar		Ash (%)	GCV (MJ/Kg)	K (g/kg)	Na (mg/kg)	Ca (g/kg)	Mg (g/kg)
**0**	Ala	NM	5.5 ± 0.2c	17.4 ± 0.0ab	18.3 ± 0.6bc	127.8 ± 12.4e	3.3 ± 0.0e	3.3 ± 0.2cd
		M	5.5 ± 0.4c	17.4 ± 0.0ab	11.9 ± 5.2c	134.9 ± 6.6de	4.4 ± 0.2cd	4.2 ± 0.2ab
	Kan	NM	5.8 ± 0.6bc	17.3 ± 0.1b	25.4 ± 1.4a	177.6 ± 14.1bc	4.6 ± 0.1cd	3.1 ± 0.0cd
		M	6.1 ± 0.1bc	16.9 ± 0.0c	24.3 ± 0.5ab	164 ± 19.5cd	4.9 ± 0.0c	3.7 ± 0.1bc
	Per	NM	6.0 ± 0.2bc	17.5 ± 0.0ab	24.9 ± 1.8ab	207.5 ± 14.8b	4.7 ± 0.2cd	4.0 ± 0.1abc
		M	6.1 ± 0.5bc	17.4 ± 0.2ab	25.9 ± 2.8a	292.7 ± 12.4a	4.2 ± 0.0d	3.6 ± 0.1bc
	Bw	NM	7.7 ± 0.0a	17.3 ± 0.0b	21.6 ± 0.9ab	20.9 ± 0.6f	6.6 ± 0.2b	2.6 ± 0.1d
		M	6.9 ± 0.2ab	17.6 ± 0.0a	22.6 ± 3.0ab	30.3 ± 0.9f	6.6 ± 0.3b	4.2 ± 0.3ab
	Sum	NM	7.7 ± 0.7a	16.8 ± 0.2c	14.3 ± 0.6c	20.5 ± 2.2f	9.1 ± 0.2a	4.0 ± 0.5abc
		M	6.8 ± 0.3ab	17.3 ± 0.1ab	14.8 ± 0.1c	35.9 ± 6.6f	6.9 ± 0.4b	4.6 ± 0.5a
**1**	Ala	NM	5.8 ± 0.3b	17.4 ± 0.1cde	17.9 ± 0.4c	154.8 ± 13.6c	3.2 ± 0.2 g	3.6 ± 0.0cd
		M	4.7 ± 0.1c	17.3 ± 0.1de	18.9 ± 0.5bc	445.4 ± 10.7a	4.3 ± 0.1e	5.6 ± 0.1a
	Kan	NM	5.9 ± 0.3b	17.8 ± 0.1a	23.5 ± 1.8a	85.3 ± 7.6d	4.3 ± 0.0e	3.2 ± 0.2d
		M	5.9 ± 0.1bc	17.3 ± 0.1de	21.8 ± 0.9ab	130.4 ± 15.4c	4.5 ± 0.0e	3.6 ± 0.4cd
	Per	NM	5.1 ± 0.3bc	17.5 ± 0.1bcd	20.4 ± 1.1bc	131.4 ± 29.2c	3.5 ± 0.3fg	3.2 ± 0.2d
		M	5.7 ± 0.3b	17.7 ± 0.1ab	23.1 ± 1.4a	318.3 ± 17.2b	3.8 ± 0.1f	3.4 ± 0.1cd
	Bw	NM	5.8 ± 0.1b	17.6 ± 0.0abc	18.2 ± 0.4bc	52.4 ± 7.5de	7.6 ± 0.2c	3.4 ± 0.0cd
		M	7.3 ± 0.0a	17.8 ± 0.1a	25.0 ± 2.2a	77.9 ± 6.7d	6.4 ± 0.2d	3.9 ± 0.1bc
	Sum	NM	7.1 ± 0.5a	16.9 ± 0.0f	18.2 ± 2.1bc	33.5 ± 2.0e	9.5 ± 0.1a	3.5 ± 0.0cd
		M	7.7 ± 0.2a	17.3 ± 0.0e	17.4 ± 0.6c	51.1 ± 2.2de	8.2 ± 0.0b	4.2 ± 0.3b
**110**	Ala	NM	6.3 ± 0.2c	17.1 ± 0.1cd	19.7 ± 0.3bc	212.4 ± 28.0c	5.2 ± 0.3e	4.6 ± 0.4a
		M	5.8 ± 0.6c	17.2 ± 0.1bcd	23.0 ± 2.9abc	290.9 ± 14.7a	4.7 ± 0.3e	4.7 ± 0.4a
	Kan	NM	6.2 ± 0.4c	17.5 ± 0.0a	22.1 ± 0.8abc	60.8 ± 6.7ef	3.4 ± 0.1 g	2.8 ± 0.0c
		M	5.3 ± 0.3c	17.4 ± 0.1ab	21.4 ± 0.9abc	100.2 ± 14.6d	3.9 ± 0.0fg	3.5 ± 0.5bc
	Per	NM	5.9 ± 0.3c	17.3 ± 0.2abc	22.5 ± 0.6abc	248.5 ± 2.0bc	6.0 ± 0.5d	2.9 ± 0.2c
		M	6.0 ± 0.2c	17.2 ± 0.0bcd	25.3 ± 0.8a	264.0 ± 16.1ab	4.6 ± 0.1ef	3.0 ± 0.1c
	Bw	NM	6.5 ± 0.3bc	17.4 ± 0.0ab	22.2 ± 1.2abc	46.3 ± 0.3ef	8.0 ± 0.3b	3.1 ± 0.1c
		M	6.7 ± 0.4bc	17.5 ± 0.1a	24.3 ± 2.8ab	64.3 ± 6.3def	6.8 ± 0.1c	4 ± 0.1ab
	Sum	NM	7.7 ± 0.4b	16.4 ± 0.0e	14.7 ± 1.6d	29.1 ± 4.5f	10.8 ± 0.4a	4.2 ± 0.1a
		M	9.6 ± 0.6a	17.1 ± 0.0d	19.2 ± 2.3cd	78.5 ± 0.2def	7.5 ± 0.1bc	4.2 ± 0.0a

The ash contents in the two highland cultivars were higher than those in the three lowland cultivars, irrespective of inoculation. The ash contents in the inoculated cultivars were similar to those in the non-inoculated treatments, except Ala and Kan, which decreased by 17.9%, 5.7% and by 8.9%, 12.3% compared with the corresponding non-inoculated treatment with 1 and 10 mg/kg of added Cd, respectively, and Bw and Sum, which increased by 26.4%, 7.9% and 3.6%, 25.3% compared with the corresponding non-inoculated treatment with 1 and 10 mg/kg of added Cd, respectively. The GCV of inoculated Kan decreased by 1.9% and 2.8% compared to that of the non-inoculated treatment at 0 and 1 mg/kg Cd addition levels, respectively. The values of Bw with AMF symbiosis were 1.9% higher than the values without AMF symbiosis under 0 mg/kg, and the GVC of inoculated Sum were higher than those of the non-inoculated treatment at every Cd level, increasing by 3.0%, 2.3%, and 3.8% at correspondingly increased levels of Cd addition. However, AMF inoculation had no significant effect on the GVC of other cultivars.

The Na, Ca, and Mg concentrations varied in different cultivars and inoculation treatments. The Na concentrations of lowland cultivars maintained at 60–450 mg/kg were higher than those of the two highland cultivars maintained at 20–80 mg/kg. As the soil Cd increased, the concentration of Na in Bw and Sum increased, irrespective of inoculation, and the same trend was detected in Ala without AMF symbiosis; however, an opposite trend was observed in Kan. The Na concentrations in Ala, Bw, and Sum in 1 and 10 mg/kg Cd soil and in Kan and Per in 1 mg/kg Cd soil with AMF symbiosis were higher than those in the non-inoculated treatments. The Ca concentrations in Bw and Sum (6–11 g/kg) were higher than those in the three lowland cultivars (3–6 g/kg). With AMF symbiosis, the Ca concentrations increased in Ala in 0 and 1 mg/kg Cd soil; however, the Ca concentrations decreased in Per in 10 mg/kg Cd soil and in Bw and Sum with RI at all Cd additional levels compared with those in the corresponding non-inoculated treatments. The Mg concentrations in Kan and Per decreased with increasing soil Cd, while they increased in Ala and Bw without AMF and remained stable in Bw with AMF and in Sum regardless of inoculation. The AMF symbiosis greatly increased Mg concentrations in Ala with 0 and 1 mg/kg Cd, Bw with 0 and 10 mg/kg Cd levels, and Sum with 1 mg/kg Cd.

### PCA of the effects of AMF on bioenergy quality-related factors

**Figure 5:**
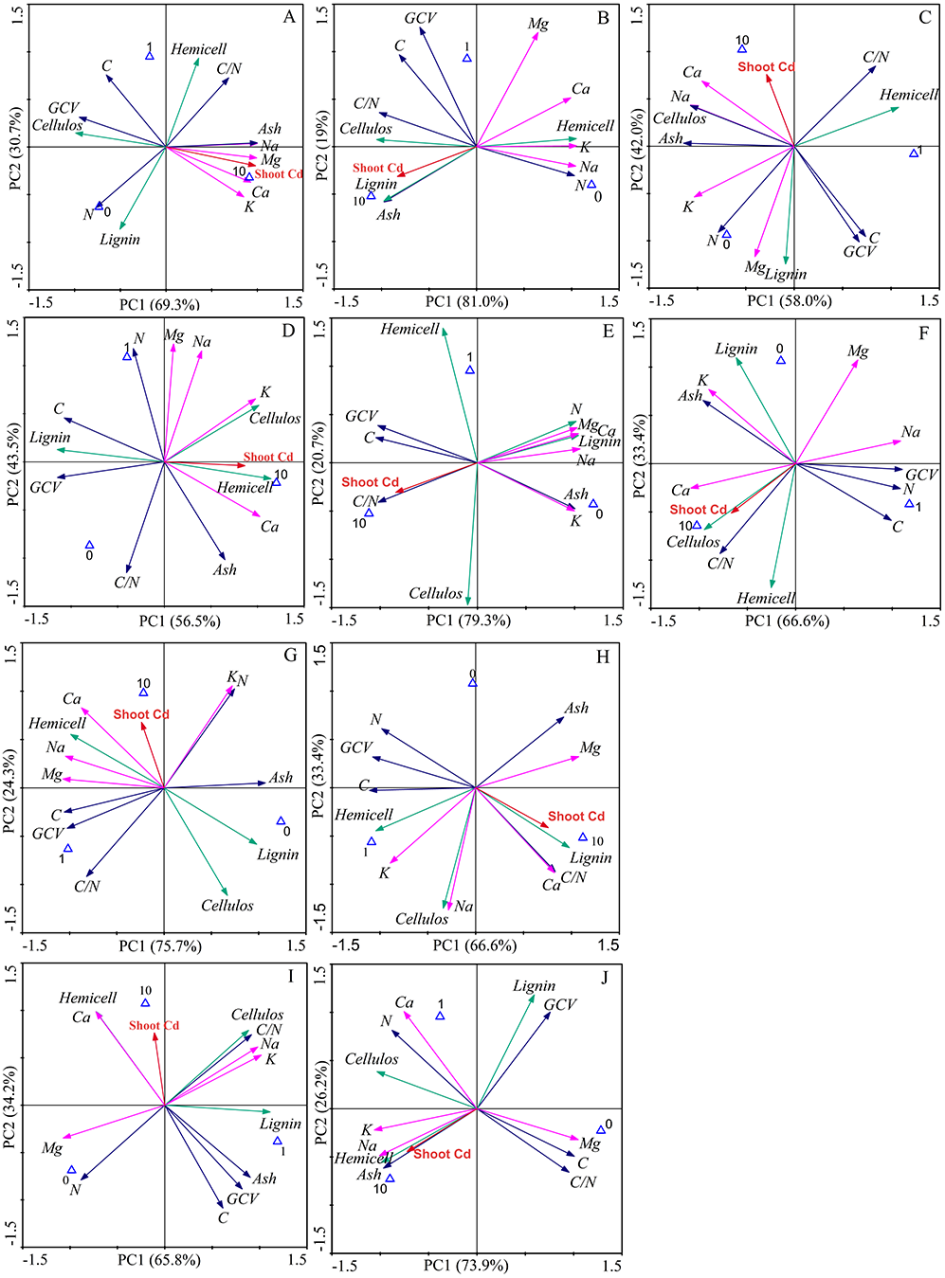
Principle component analysis of bioenergy quality-related factors of five switchgrass cultivars with NM and M treatments under Cd stress. (A, D) represent non-inoculated with AMF (NM) and inoculated with AMF (M) treatments in Ala. (B, E) represent NM and M treatments in Kan. (C, F) represent NM and M treatments in Per. (G, I) represent NM and M treatments in Bw. (H, J) represent NM and M in treatments Sum. Different colors of arrows among the factors represent corresponding bioenergy quality (green: cellulose quality; pink: alkaline elements, blue: other factors). The blue triangle represent the Cd addition level (0, 1, 10 mg/kg).

The first two components explained 100% of the total data variability of the bioenergy quality under three concentrations of Cd, regardless of inoculation (Fig. 5). For the Ala, Kan, Per, Bw and Sum cultivars, 69.3%, 81.0%, 58%, 75.7%, and 66.6% of the variability, respectively, was explained by PC1, and 30.7%, 19%, 42%, 24.3%, and 33.4% of the variability, respectively, was explained by PC2. For the corresponding inoculated treatments, PC1 represented 56.5%, 79.3%, 66.6%, 65.8%, and 73.9% of the variability, respectively, and PC2 represented 43.5%, 20.7%, 33.4%, 34.2%, and 26.2% of the variability, respectively.

The loading values of bioenergy quality-related factors represented the correlation with the corresponding PCs. The results showed that the AMF inoculation changed the factors’ correlations under Cd stress, although the PCs varied for different cultivars (Tables S2–S3). AMF symbiosis changed the factor loading values in five ways. (1) From low to high correlation in PC1 or PC2: the values of C, N, C/N, and ash of Ala, the N and C/N of Kan, the GCV of Kan, the C/N and K of Bw, and the C/N of Sum were higher than 0.8 with AMF symbiosis and lower than 0.8 without AMF. (2) From high to low correlation in PC1 or PC2: this effect was only found in the highland cultivars, including in the GCV of Bw and Sum and the hemicellulose of Bw, in which the loading values were lower than 0.8 after AMF inoculation. (3) The transformation between positive and negative loading values in the PCs: the loading values of the lignin of Ala, the lignin and ash of Kan, the lignin and ash of Bw, and the ash of Sum changed from negative to positive values with AMF symbiosis, whereas the lignin, Na, and Mg of Per, the Mg of Bw, and the C/N of Sum represented the opposite trend. (4) The correlation transformation between PC1 and PC2: the hemicellulose of Ala, the Mg of Kan, and the cellulose and Na of Sum that were PC2 in the absence of AMF were transformed into PC1 with the presence of AMF; reverse trends were obtained for the Na and Mg of Ala and the hemicellulose and lignin of Kan. In addition, (3) and (4) were observed simultaneously for the GCV of Kan, the hemicellulose of Per, the C of Bw and the lignin of Sum with AMF symbiosis. However, regardless of inoculation, the loading values of K and Ca in the highland cultivars were consistently correlated, and the cellulose of Bw and the Ca of the highland cultivars showed poor associations with the PCs.

## Discussion

### Biomass and P

Biomass as the key factor to evaluate energy grass productivity is also an index of the heavy metal tolerance of plants. In this experiment, the biomasses of five cultivars first increased and then decreased with increasing soil Cd, irrespective of inoculation. This trend indicated that the amount of Cd promoted switchgrass growth, as reported in *Pennisetum americanum* and *Vetiveria zizanioides* (Aibibu et al., 2010; Zhang et al., 2010). In addition, the growth of inoculated switchgrass was much better than that of non-inoculated plants, irrespective of the cultivar. The biomass mycorrhizal response varied among different cultivars, especially in Sum in which the highest shoot %MGR reached 140 and the highest root %MGR reached 117, while the highest %MGRs in the shoots and roots of other cultivars were 107 and 44, respectively (Table 1). Hence, the enhanced growth of switchgrass by AMF varied among cultivars. In addition, a low concentration of Cd further strengthened the growth promotion by AMF, especially in Ala, Kan and Bw. Most researchers have reached a consensus that AMF growth enhancement contributes to the increased P concentration (Chen et al., 2007; De Andrade et al., 2008). In the present study, the P concentrations in all cultivars increased significantly with AMF presence, but the promotion effects on plant growth from the additional P due to AMF inoculation were varied. The shoot %MGR of the five grasses did not show any relationship with the %MPR, but in the roots, a negative correlation was observed between %MGR and %MPR at all Cd levels (*R*^2^ = 0.4855, Table 1). This outcome was contrary to that of Zhang, Chen & Ohtomo (2015) who reported that the %MGR of *Medicago sativa* inoculated with RI showed the same increasing trend as %MPR as soil Cd increased. The %MGR and %MPR did not exhibit any regular variation patterns in *Taraxacum platypecidum* and *Cynodon dactylon* at different Cr addition levels (Wu et al., 2014). Thus, these differences can be explained in part by the plant and contaminant species.

### Cd concentration and extraction

The results of this study indicate that most of the Cd accumulated in the belowground portions of all cultivars except Sum. It was known that AMF help to chelate Cd in plant vacuoles and cell walls, and its arbuscular structure or extraradical mycelium result to Cd accumulation in plant belowground organs (Yao et al., 2014; Li et al., 2016). However, the %MCdRs were negative in the lowland types and positive in the highland types, except when the soil Cd was 0 mg/kg. Previous studies have found that AMF has contrast strategy for heavy metal absorption for different cultivars. Some researchers found that inoculation with AMF alleviate Cd toxicity by decreasing contaminants accumulation in *oryza sativa*, irrespective of shoot and root (Li et al., 2016; Luo et al., 2017), as observed in Ala, Kan in present study (Fig. 3). Besides the increased biomass with inoculation diluting Cd concentration in plants, AMF could decrease Cd bio-available by secreting glomalin binding Cd, increasing soil pH, and mobilized toxic metals in extraradical mycelium to diminish Cd absorption (González-Chávez et al., 2004; Wang et al., 2012; Wu et al., 2015). Whereas some researchers found that AMF increased Cd concentrations in shoots of *Solanum nigrum, Lotus japonicas*, *Phragmites australis* (Liu et al., 2015; Zhang, Chen & Ohtomo, 2015; Wang et al., 2017). In addition, the shoot and root Cd concentrations of Bw and Sum were increased, the %MCdR of Bw reached 252 and 156 in the roots under 1 and 10 mg/kg, respectively, and the %MCdR in the shoots and roots of Sum was 84 in 1 mg/kg Cd soil, which was much higher than those in other cultivars (Table 1). These results are likely to be related to the root architecture of these two types. There were more fine roots in highland cultivars, and AMF furtherly promoted their Cd contact efficiency by having longer and more branches, and sequenced Cd in plant cell wall and vacuole (Graaff et al., 2013; Li et al., 2016). The increased shoot Cd might related to the increasing root Cd concentration and the Cd translocation ability in Sum. Moreover, Dietterich, Gonneau & Casper (2017) reported AMF had little consequence on Cd uptake in field soils. Therefore, the effects of AMF symbiosis on plant Cd absorption and accumulation might depend on plant species, even the cultivar.

In addition, the different mycorrhizal responses to biomass and Cd concentration in these cultivars lead to different Cd extraction. Symbiosis with RI greatly increased the total and root Cd extracted amount of Bw at 1 and 10 mg/kg Cd addition levels, resulting in the amount that were much higher than those in other cultivars at different Cd levels. The extracted Cd in Ala were slightly less than those in Bw, but the shoot amount of extracted Cd in Ala were higher and smeltable at the 1 and 10 mg/kg Cd addition levels, respectively (Fig. 4). Therefore, inoculated Bw could be planted in seriously Cd-polluted areas to phytostabilize the contaminants, and inoculated Ala is suitable for planting in less Cd-polluted areas.

### C, N, C/N

In this study, Cd significantly affected the C, N, and C/N contents, which are factors of anaerobic digestion in the production of biogas (*P* < 0.05, Table S1). Cd influences anaerobic digestion and biogas production because it disrupts enzyme function and structure. Cd binds to metal ions with thiol and other groups and replaces naturally occurring metals in the enzyme prosthetic groups (Mudhoo & Kumar, 2013). Xu et al. (2017) reported that low concentrations of Cd (0.1 mg/g VSS) benefited hydrolysis and acidogenesis and increased the microbial diversity, the abundance of functional microbes, and the activities of key enzymes during the anaerobic fermentation of sludge, whereas higher concentrations of Cd (10 mg/g VSS) inhibited these factors. Lee et al. (2017) used sunflowers harvested in a heavy metal-contaminated site (3.98 mg Cd) for anaerobic digestion and found that the continuously stirred-tank reactor remained stable for 20 days and that the microorganisms in the reactor did not suffer from extraneous toxic substances. Hence, substrates with exogenous and endogenous Cd can be used for sustainable biogas production. In the present study, the AMF inoculation significantly influenced the C, N, and C/N contents in switchgrass. Currently, no published studies have investigated biomass inoculated with AMF for anaerobic digestion.

AMF promoted plant photosynthesis and carbon assimilation, with the host plant providing a carbon source to AMF in return (He et al., 2017). However, inoculated AMF had no effect on total C contents in the present study except in Sum at each Cd level and Per in 1 mg/kg Cd soil, perhaps because of the decreased shoot Cd concentrations in these cultivars with AMF symbiosis, which resulted in less damage to the switchgrass photosynthetic organs, and the assimilated C satisfied the demand for plant growth. Carbon not only offered materials and energy to the symbiont but also regulated the nutrition dynamic equilibrium between AMF and the plant (Campossoriano & Segundo, 2011). Increased N contents were observed in Per, Bw and Sum at each Cd addition level, while they decreased or remained stable in Ala (Table 2). A possible explanation for these results might be the different N demand in these cultivars. The enhancement of plant growth and Cd tolerance by AMF might be based on sufficient N, since the preceding studies found that AMF have high N requirements, insufficient N limits AMF development in poor soil and AMF colonization is promoted by N addition (Grman & Robinson, 2013; Hodge & Díaz, 2010; Liu et al., 2017).

The material used for fermentation suitable for regulating the C/N is 16–20 (Mata-Alvarez, 2002). In this experiment, the C/N in all cultivars was between 25 and 30, except in Ala in which the C/N ranged from 30 to 35 because the N contents were lower in Ala than those in other cultivars at different Cd levels, irrespective of inoculation. The lower N content leads to C surplus and organic acid accumulation that would not contribute to methane fermentation. Therefore, the influence of switchgrass cultivar on C/N was more significant than inoculation (Table S1). It suggests that a certain amount of nitrogen-rich substrates, such as excrement of livestock and humans, should be added to adjust the C/N in switchgrass for methane fermentation.

### Hemicellulose, cellulose and lignin

The hemicellulose, cellulose and lignin in plant cell walls are combined by ester bonds, ether bonds and glycosides. Lignin affected the production of methane and bioethanol by obstructing the decomposition efficiency of cellulose (Monlau et al., 2012). Lignin is the physical barrier against pathogens and is synthesized by the phenylpropanoid pathway, which begins with the cinnamic acid synthesis reaction catalyzed by phenylalanine ammonia-lyase from phenylalanine (Hisano, Nandakumar & Wang, 2009; Jeong et al., 2012). Previous studies found that Cd increases PAL activity in different plants and organs (Yang et al., 2015; Loix et al., 2017) because lignification limits the root exposure to Cd and reinforces the plant defense system (Fingerteixeira et al., 2010). In the present study, the lignin content was significantly affected by Cd (*P* < 0.05, Table S1), and the lignin content of Ala, Per and Bw after treatment with 0 mg/kg Cd was increased compared with the levels obtained after treatment with 1 and 10 mg/kg Cd in soil without AMF symbiosis. This result might be due to the destruction of the root structure by Cd, which can block shoot growth and development and thereby limit lignin biosynthesis. Inoculation with AMF also increased the activity of PAL and the expression of the L-phenylalanine ammonia-lyase gene (Zhu et al., 2015), Liu et al. (2014) reported that the lignin content of popular seedlings was increased with AMF inoculation, which was consistent with the trend we observed in Kan in the presence of 1 mg/kg Cd. However, the lignin contents of Bw with 0 mg/kg Cd, Per with 1 mg/kg Cd and Ala with 1 and 10 mg/kg Cd combined with AMF symbiosis were all lower than those in the corresponding non-inoculated treatments (Table 2), possibly because the Cd concentration in the shoots decreased with AMF symbiosis, alleviating shoot lignification. According to these results, we can infer that the biosynthesis of lignin was affected by AMF inoculation and Cd addition. However, regulating the synthesis of lignin to protect plants from fungal invasion or Cd toxicity might depend on the plant characteristics of Cd tolerance and affinity for mycorrhizal fungi. In addition, it is unknown what role PAL played in lignin synthesis with Cd addition and AMF symbiosis, so further study is needed to determine the mechanism to produce lower lignin biomass.

### Ash, GCV, alkalis elements

The bioenergy quality evaluates the conversion potential of energy grass into biofuel, bioethanol and methane (Schmer et al., 2008; Koçar & Civaş, 2013; Wu et al., 2015; Kou et al., 2017). In general, biomass should contain lower ash and alkali metal content to protect the boiler from coking and blocking (Demirbas, 2001). Plant ash is instituted by the mineral elements and their oxides, and the contents of ash reflect the plant enrichment ability of mineral elements. The ash contents in the three inoculated lowland cultivars remained stable or decreased at all Cd addition levels, while the ash contents in the highland cultivars with AMF symbiosis were higher than those in the corresponding non-inoculated and lowland cultivars at the 1 and 10 mg/kg Cd addition levels (Table 3). These results were in accord with the Cd concentrations observed in this study, indicating that the composition of the ash was affected by the Cd absorption by the shoots. In addition, the Ca concentrations in highland cultivars were higher than those in lowland cultivars over all Cd addition levels (Table 3). Hence, the lowland cultivars had lower ash resulting from the lower Cd and Ca accumulation in their shoots. K is the most important alkali metal for biomass combustion since it reduces the ash dissolution temperature, leading to slagging and pollution in the combustor (Miles et al., 1996), but in the present study, neither Cd nor inoculation had any effect on the K concentration in any cultivar. Although the Na and Mg concentrations with AMF symbiosis were higher than in non-inoculated plants and they might have synergistic effects with Cd absorption in all cultivars, they did not correlate with the ash content (Sarwar et al., 2010).

The GCV was the direct index for bioenergy that was related to C assimilation ability in the photosynthetic process. In the present study, although the GCV in inoculated Sum was higher than that in the corresponding non-inoculated plants at every Cd level and the GCV in inoculated Kan was lower than that the non-inoculated plants in 0 and 10 mg/kg Cd soil, the values of the other cultivars remained the same regardless of AMF inoculation. The decreased GCV in Kan under 1 mg/kg Cd addition, it may result from the C offered by the host participating in other metabolic pathways for the germination of spores and the development of mycelium since total C contents in Kan were higher than those in the non-inoculated treatment and its root colonization was higher than other Cd level (Fig. S1). Liu et al. (2014) reported that AMF promoted GCV in popular seedlings, which was similar to the trend in non-inoculated Sum. In general, AMF increases plant photosynthesis, accelerates C assimilation, and promotes plant growth to receive more C from most host plants (Amna et al., 2015; Liu et al., 2015). In our study, the switchgrass was already in the flowering period, and the different C supply and demand of the plants in different growth periods may lead to the different GCVs. Therefore, selecting a proper harvest time is critical for assuring biomass GCVs.

Previous studies have characterized the mechanism through which Cd tolerance is increased by mineral elements and reactive oxygen species through a PCA with large amounts of data (Huang, Wang & Ma, 2017). In our study, the correlations of bioenergy quality-related factors to the principle components varied between cultivars, although the K and Ca in the lowland cultivars clustered as positive loading values for PC1 under both NM and M treatments, perhaps because the lowland cultivars regulate Ca and K absorption to protect against Cd stress, regardless of inoculation (Sarwar et al., 2010). The decreased Ca loading values in the highland cultivars (<0.8) might be due to their inherently higher Ca concentrations.

## Conclusion

In this experiment AMF enhanced the growth and P concentrations of the five cultivars. However, AMF inoculation influenced different strategies regarding Cd absorption and extraction in lowland and highland cultivars. AMF symbiosis decreased the concentration of Cd in the shoots and roots of Ala and Kan, and increased Cd concentrations in the roots of Bw and the shoots of Sum. Although AMF inoculation changed the Cd concentrations in these cultivars and allowed Bw to extract the highest quantity of Cd in the roots, the total extracted Cd in the lowland cultivars was not significantly affected by the presence or absence of AMF (except for Ala, Per with 1 mg/kg Cd). The reduced Cd and Ca contents in the shoots of lowland cultivars might lead to lower ash contents in these cultivars. AMF promoted C content and GCV in Sum, but the increased levels of ash and decreased biomass resulted in low phytoremediation efficiency and bioenergy quality. Ala showed decreased lignin content and increased Cd extraction in the shoots under low Cd stress with AMF symbiosis. Therefore, inoculation with AMF provided the highest yield and the finest quality biomass in switchgrass, increasing the phytoremediation potential in Cd-polluted soils. The PCA comprehensively reflects the correlation of bioenergy quality-related factors in switchgrass with Cd addition and AMF symbiosis.

##  Supplemental Information

10.7717/peerj.4425/supp-1 Data S1 Raw data

10.7717/peerj.4425/supp-2 Figure S1 The root colonization of five cultivars under three Cd addition levels The letters in the column with the same lines represented the significantly difference at 0.05 levels of the same cultivar under three Cd levels.

10.7717/peerj.4425/supp-3 Table S1 ANOVA analysis the effect of Cultivar, Inoculated and Cd addition on the Contents of C, N, C/N, hemicellulose, cellulose, lignin, ash, GCV, K, Na, Ca, Mg of switchgrass The *** represented *P* < 0.001, ** represented *P* < 0.01, *represented *P* < 0.05.

10.7717/peerj.4425/supp-4 Table S2 Loading for PC1 and PC2 of principal component analysis of the bioenergy factors and alklin elements with non-inoculated with AMF of five cultivars Eigenvalues in bold >0.80 indicate high association for interpretation of the principal component analysis.

10.7717/peerj.4425/supp-5 Table S3 Loading for PC1 and PC2 of principal component analysis of the bioenergy factors and alkaline elements with inoculated with AMF of five cultivars Eigenvalues in bold >0.80 indicate high association for interpretation of the principal component analysis.

## Additional Information and Declarations

### Data Availability

The following information was supplied regarding data availability: The raw data has been provided as a Supplemental File.
