# Supplementary figures and images for: The enhancement by arbuscular mycorrhizal fungi of the Cd remediation ability and bioenergy quality-related factors of five switchgrass cultivars in Cd-contaminated soil
Source: PeerJ. 2018 Mar 6;6:e4425. doi: 10.7717/peerj.4425 (PMC5844250; doi:10.7717/peerj.4425)

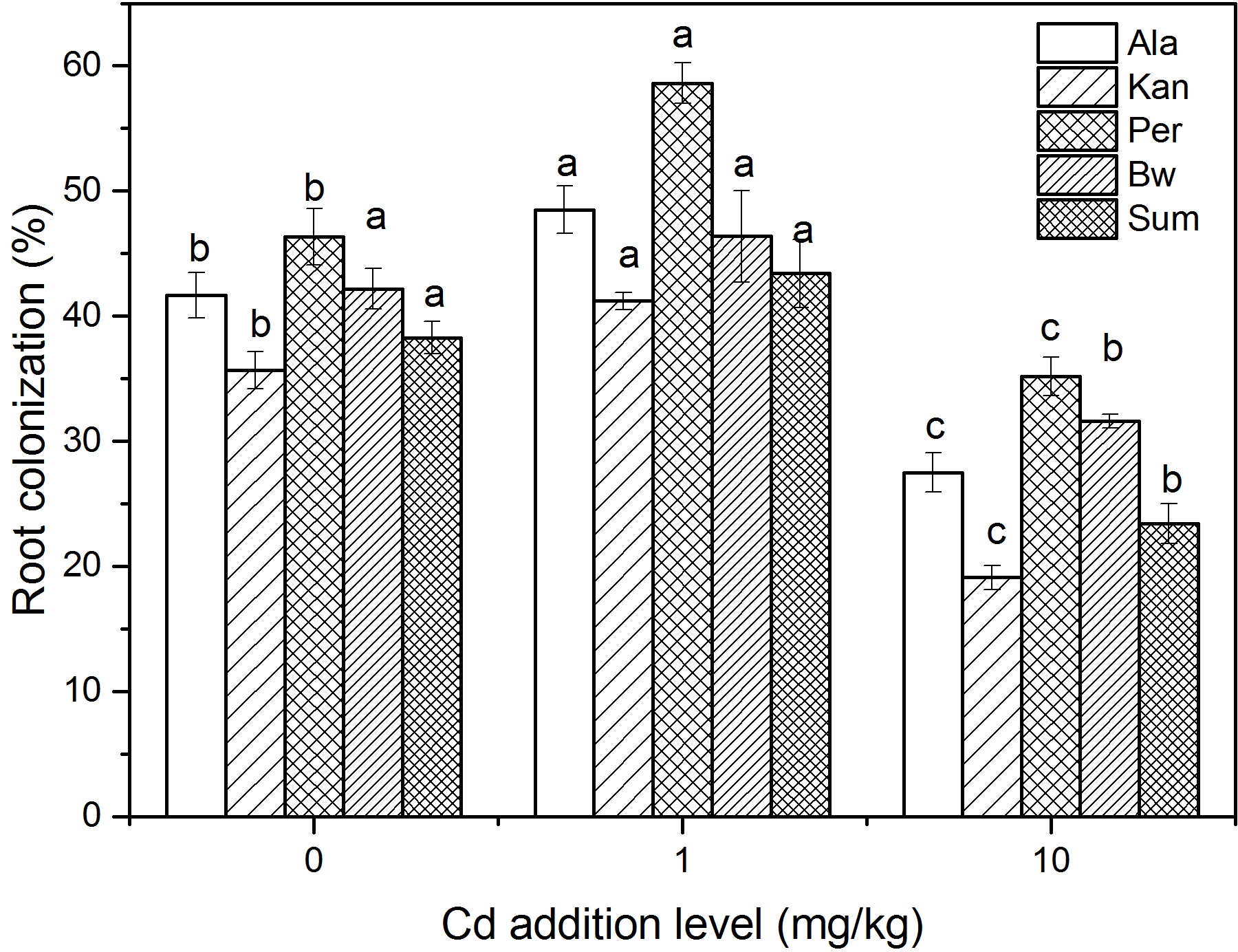

Supplement: Figure S1 — The letters in the column with the same lines represented the significantly difference at 0.05 levels of the same cultivar under three Cd levels. [file peerj-06-4425-s002.png]
