# Supplementary material for: The enhancement by arbuscular mycorrhizal fungi of the Cd remediation ability and bioenergy quality-related factors of five switchgrass cultivars in Cd-contaminated soil
Source: PeerJ. 2018 Mar 6;6:e4425. doi: 10.7717/peerj.4425 (PMC5844250; doi:10.7717/peerj.4425)
Supplement: Table S1 — The *** represented P < 0.001, ** represented P < 0.01, *represented P < 0.05. [file peerj-06-4425-s003.docx]

|  | Cultivar (C) | Inoculation  (I) | Cd level  (Cd) | C × I | C × Cd | I × Cd | C × Cd ×I |  |
| --- | --- | --- | --- | --- | --- | --- | --- | --- |
| C (%) | | **0.000^***^** | **0.000^***^** | **0.011^*^** | 0.171 | 0.383 | 0.315 | 0.511 |
| N (%) | | **0.000^***^** | **0.007^**^** | **0.000^***^** | 0.240 | **0.000^***^** | **0.007^**^** | **0.000^***^** |
| C/N | | **0.000^***^** | **0.001^**^** | **0.010^**^** | 0.324 | 0.180 | 0.130 | 0.498 |
| Hemicellulose (%) | | **0.003^**^** | 0.216 | **0.041^*^** | 0.985 | 0.677 | 0.661 | 0.999 |
| Cellulose (%) | | **0.001^**^** | 0.532 | 0.071 | 0.786 | 0.157 | 0.057 | 0.611 |
| Lignin (%) | | **0.013^***^** | 0.073 | **0.023^*^** | 0.159 | 0.066 | 0.230 | 0.053 |
| Ash (%) | | **0.000^***^** | 0.663 | **0.008^**^** | 0.108 | **0.041^*^** | 0.269 | **0.007^**^** |
| GCV(MJ/kg) | | **0.000^***^** | **0.023^*^** | **0.000^***^** | **0.000^***^** | **0.000^***^** | 0.596 | 0.115 |
| K (g/kg) | | **0.000^***^** | 0.127 | 0.340 | 0.166 | **0.012^*^** | 0.094 | 0.380 |
| Na (mg/kg) | | **0.000^***^** | **0.000^***^** | **0.000^***^** | **0.000^***^** | **0.000^***^** | **0.000^***^** | **0.000^***^** |
| Ca (g/kg) | | **0.000^***^** | **0.000^***^** | **0.000^***^** | **0.000^***^** | **0.000^***^** | **0.000^***^** | **0.000^***^** |
| Mg (g/kg) | | **0.000^***^** | **0.000^***^** | 0.893 | 0.001 | **0.000^***^** | 0.123 | **0.007^**^** |
