# Supplementary material for: The enhancement by arbuscular mycorrhizal fungi of the Cd remediation ability and bioenergy quality-related factors of five switchgrass cultivars in Cd-contaminated soil
Source: PeerJ. 2018 Mar 6;6:e4425. doi: 10.7717/peerj.4425 (PMC5844250; doi:10.7717/peerj.4425)
Supplement: Table S2 — Eigenvalues in bold >0.80 indicate high association for interpretation of the principal component analysis. [file peerj-06-4425-s004.docx]

|  | Ala  PC1 PC2 | | Kan  PC1 PC2 | | Per  PC1 PC2 | | Bw  PC1 PC2 | | Sum  PC1 PC2 | |
| --- | --- | --- | --- | --- | --- | --- | --- | --- | --- | --- |
| C (%) | -0.667 | 0.745 | -0.783 | 0.622 | -0.660 | 0.751 | **0.985** | -0.171 | **1.000** | 0.015 |
| N (%) | -0.756 | -0.655 | **0.982** | -0.190 | 0.669 | 0.743 | -0.698 | 0.716 | **0.879** | -0.476 |
| C/N | 0.656 | 0.755 | **-0.978** | 0.211 | -0.730 | -0.684 | 0.769 | -0.639 | -0.746 | 0.666 |
| Hemicellulose (%) | 0.333 | **0.943** | **0.998** | 0.070 | **-0.939** | -0.343 | **0.918** | 0.396 | 0.943 | 0.332 |
| Cellulose (%) | **-0.992** | 0.124 | **-1.000** | 0.025 | **0.942** | -0.336 | -0.627 | -0.779 | 0.322 | 0.947 |
| Lignin (%) | -0.494 | **-0.870** | **-0.915** | -0.404 | 0.069 | **0.998** | **-0.912** | -0.410 | **-0.875** | 0.483 |
| Ash (%) | **0.999** | 0.042 | **-0.915** | -0.403 | **1.000** | -0.004 | **-1.000** | -0.003 | **-0.834** | -0.551 |
| GCV(MJ/kg) | **-0.956** | 0.295 | -0.576 | **0.817** | -0.603 | 0.798 | **0.957** | -0.290 | **0.968** | -0.252 |
| K (g/kg) | **0.866** | -0.500 | **1.000** | 0.003 | **0.883** | 0.470 | -0.668 | 0.744 | **0.815** | 0.579 |
| Na (mg/kg) | **1.000** | 0.000 | **0.973** | -0.230 | **0.918** | -0.397 | **0.984** | 0.180 | 0.320 | **0.947** |
| Mg (g/kg) | **0.993** | -0.118 | 0.589 | **0.808** | 0.360 | **0.933** | **0.999** | 0.049 | **-0.976** | -0.217 |
| Ca (g/kg) | **0.939** | -0.345 | **0.944** | 0.330 | **0.808** | -0.589 | 0.799 | 0.601 | -0.744 | 0.668 |
