# Supplementary material for: The enhancement by arbuscular mycorrhizal fungi of the Cd remediation ability and bioenergy quality-related factors of five switchgrass cultivars in Cd-contaminated soil
Source: PeerJ. 2018 Mar 6;6:e4425. doi: 10.7717/peerj.4425 (PMC5844250; doi:10.7717/peerj.4425)
Supplement: Table S3 — Eigenvalues in bold >0.80 indicate high association for interpretation of the principal component analysis. [file peerj-06-4425-s005.docx]

|  | Ala  PC1 PC2 | | Kan  PC1 PC2 | | Per  PC1 PC2 | | Bw  PC1 PC2 | | Sum  PC1 PC2 | |
| --- | --- | --- | --- | --- | --- | --- | --- | --- | --- | --- |
| C (%) | **-0.970** | 0.245 | **-0.987** | 0.158 | **-0.896** | 0.444 | 0.565 | **-0.825** | **-0.929** | -0.369 |
| N (%) | -0.414 | **0.910** | **0.956** | 0.293 | **-0.980** | 0.197 | -0.784 | -0.620 | **0.802** | 0.597 |
| C/N | -0.225 | **-0.974** | **-0.950** | -0.313 | 0.708 | 0.706 | **0.814** | 0.581 | **-0.879** | -0.477 |
| Hemicellulose (%) | **1.000** | -0.011 | -0.347 | **0.938** | 0.234 | **0.972** | -0.660 | 0.751 | **0.926** | -0.377 |
| Cellulose (%) | **0.812** | 0.584 | -0.082 | **-0.997** | **0.854** | 0.521 | 0.785 | 0.619 | **0.955** | 0.295 |
| Lignin (%) | **-0.999** | -0.035 | **0.982** | 0.187 | 0.534 | **-0.846** | **0.999** | -0.051 | -0.565 | **0.825** |
| Ash (%) | 0.681 | -0.732 | **0.950** | -0.312 | **0.867** | -0.499 | **0.824** | -0.566 | **0.893** | -0.450 |
| GCV(MJ/kg) | **-0.965** | -0.264 | **-0.970** | 0.242 | **-0.999** | 0.048 | 0.745 | -0.667 | -0.721 | 0.692 |
| K (g/kg) | **0.814** | 0.581 | **0.945** | -0.328 | **0.808** | -0.590 | **0.912** | 0.411 | **0.986** | -0.166 |
| Na (mg/kg) | 0.060 | **0.998** | **0.999** | 0.043 | **-0.988** | -0.153 | **0.913** | 0.408 | **0.907** | -0.420 |
| Mg (g/kg) | -0.079 | **0.997** | **0.967** | 0.254 | -0.568 | **-0.823** | **-0.956** | -0.292 | **-0.969** | -0.249 |
| Ca (g/kg) | **0.943** | -0.332 | **0.981** | 0.195 | **0.977** | 0.214 | -0.659 | 0.752 | 0.670 | 0.742 |
